# The role of tannin-based products in mitigating enteric methane emissions in ruminant livestock: A review

**DOI:** 10.5455/javar.2026.m1023

**Published:** 2026-03-16

**Authors:** Roni Pazla, Antonius Antonius, Aslizah Mohd-Aris, Zaitul Ikhlas, Yelly Fitri, Hadriana Bansi, Yelsi Listiana Dewi, Maureen Chrisye Hadiatry, Novia Qomariyah, Sutiastuti Wahyuwardani, Yenni Yusriani, Eni Siti Rohaeni, Bachtar Bakrie

**Affiliations:** 1 Department of Nutritional Science and Feed Technology, Faculty of Animal Husbandry, Universitas Andalas, Padang, West Sumatera 25175, Indonesia; 2 Research Center for Animal Husbandry, National Research and Innovation Agency (BRIN), Cibinong, West Java 16911, Indonesia; 3 School of Biology, Faculty of Applied Sciences, Universiti Teknologi MARA, Negeri Sembilan 72000, Malaysia; 4 Graduate Program, Faculty of Animal Husbandry, Universitas Andalas, Padang, West Sumatera 25175, Indonesia; 5 Research Center for Veterinary Science, National Research and Innovation Agency (BRIN), Cibinong, West Java 16911, Indonesia

**Keywords:** Enteric methane, feed additives, feed efficiency, methanogenesis inhibition, tannins

## Abstract

Global greenhouse gas accumulation receives significant contributions from methane emitted by ruminant livestock, thereby exacerbating climate change. Tannin-based feed additives are being investigated by researchers as a potential means to alter rumen fermentation and reduce methanogenesis. The researchers build on previous studies on the impact of tannins on methane reduction in the digestive tract and investigate the biological mechanisms of tannins, which are coupled with the potential of animal feed sources. Tannins kill methanogenic archaea by reducing protozoa and altering volatile fatty acid composition. This simultaneously results in reduced methane emissions and improved feed and nitrogen utilization efficiency. As a result, animal production is made more efficient by the reduction of nitrogen excretion and the enhancement of protein metabolism. The use of tannins, essential oils, biochar, and probiotics together is being researched as a way to treat the diet. Yet there are still issues, such as the adverse effects of tannins on nutrition, inconsistent tannin supply across sources, and microbial adaptation over time. The effectiveness of tannins also varies and is connected to the plant source, concentration, and processing methods. Besides, scientists are developing encapsulation methods and selecting optimal feeding protocols to increase tannin effectiveness while minimizing unwanted effects. Future researchers must improve the administration techniques for tannins, develop more efficient delivery systems, and conduct a comprehensive assessment of how tannins affect rumen microbiome health and animal performance. Tannin application emerges as an ecological approach that serves sustainability in livestock management systems and helps environmental adaptation practices.

## 1. Introduction

An important issue that continues to be a topic of global discussion is the increase in greenhouse gas concentrations, which are the main driver of global climate change. Methane is one of the most potent greenhouse gases, 28 times more potent than CO_2_, which researchers agree will have an impact for 100 years [1]. Ruminants, including cattle, buffalo, sheep, and goats, collectively produce substantial amounts of methane because of their rumen fermentation activity. The agriculture sector accounts for approximately 16 percent of global greenhouse gas emissions, while methane is the second-largest contributor to global warming [2, 3]. Enteric fermentation produces more than 95% of the methane that ruminants generate [3].

Livestock production emits nitrous oxide (N_2_O), a powerful greenhouse gas with significant global warming potential [4]. The international Paris Agreement faces significant difficulties due to the combined effects of livestock emissions and carbon dioxide, which exacerbate global warming [5]. Analyzing atmospheric methane levels from 2014 to 2017 demonstrates the pressing need to develop sustainable methods to reduce livestock emissions, given rising methane emissions from livestock and other sources [6].

Among all strategies for reducing methane emissions from cattle operations, dietary management is the most promising approach. Plant-derived compounds known as tannins stand out as natural agents that inhibit methane formation when used with various feed additives [7, 8]. The action of tannins as methane inhibitors involves microbiological suppression of methanogens, with secondary effects on rumen microbial distribution and reduced methane production. Ruminants process two categories of tannins, which include condensed tannins (CTs) and hydrolysable tannins. The flavan-3-ol subunit compounds in CTs reduce microbial action and decrease methane emissions [3]. The combined structure of polyol units with gallic or ellagic acid-derived esters in HTs affects both microbial fermentation processes alongside protein metabolism [9]. The recent findings show that the application of condensed tannins to ruminant diets results in lower methane production rates, improved nutrient utilization by the animals, and, consequently, higher feed efficiency, while the environmental impact in their region remains unchanged [10, 11].

In contrast, the activity of tannins can be affected by various factors, including their chemical substrates, the plant from which they are derived, dosage, and processing methods [12, 13, 14]. At excessive levels, tannins can have adverse nutritional effects, mainly by decreasing feed intake and nutrient digestibility [10], and prolonged use also carries the risk of microbial adaptation, which could reduce their effectiveness [15]. This intricate situation signifies the need for a more in-depth scrutiny of the parameters concerning the application of tannins for methane mitigation.

Despite abundant studies, the literature remains disjointed. The main limitation of the previous reviews was the absence of a synthesis of the results into a unifying model that would bridge the biochemical processes, rumen microbial ecology, and relevant livestock nutrition. In addition, important factors such as economic feasibility, regulatory constraints, and barriers to on-farm adoption are often overlooked. It is necessary to address these gaps to shift towards more robust evidence, thereby advancing operational approaches to sustainable livestock production.

This review is a critical literature review on tannin-based feed additives for enteric methane reduction. To be more specific, it (i) depicts a theoretical framework which relates the tannin chemistry, microorganisms pathways and animal performance; (ii) evaluates the degree and quality of evidence of studies based on *in vitro*- and *in vivo*-methods; (iii) elaborates the problem of implementation considering economic, practical and regulatory factors; and (iv) specifies the areas of knowledge that need to be filled and the further research trails that need to be followed in the context of precision livestock feeding. This integrated strategy intends to shift the discipline from merely describing the situation to providing practical solutions for sustainable ruminant production.

## 2. Materials and Methods

### 2.1. Ethical approval

This review study did not involve any experiments on live animals, animal tissues, or human participants. All data were obtained from previously published sources; therefore, ethical approval from an institutional or national ethics committee was not required.

### 2.2. Literature search strategy

The authors conducted a systematic review of the published literature on the effects of tannin-based additives on methane emissions in ruminants. A search was conducted across databases, including Scopus, PubMed, Web of Science, and Google Scholar, covering 2015–2025. These databases provide comprehensive coverage of peer-reviewed research in animal nutrition and environmental science, and were selected for this study.

The search process incorporated specific keywords and Boolean operators to help researchers identify suitable research studies. Four specific search queries were used for this investigation, which combined “Tannins AND enteric methane” with “Tannin-based feed additives AND methanogenesis inhibition” and “Tannins AND rumen fermentation AND greenhouse gas emissions” and “Tannins AND nitrogen utilization AND ruminants.” For complete literature retrieval purposes, the investigation incorporated backward citation examination, which involved examining reference sections from chosen studies to detect supplementary relevant research.

### 2.3. Inclusion and exclusion criteria

Studies were included if they met the following criteria: (i) Mechanistic insights into rumen microbiota, methanogen inhibition, or VFA modulation, (ii) Peer-reviewed articles in English with quantitative data.

The exclusion criteria were as follows: (i) Research that downplayed the role of tannins as a method for decreasing methane production, (ii) Manuscripts that were not peer-reviewed or opinion pieces, and (iii) Documents that did not present any numerical data regarding the reduction of methane or fermentation parameters.

### 2.4. Study selection process

**Figure 1.**
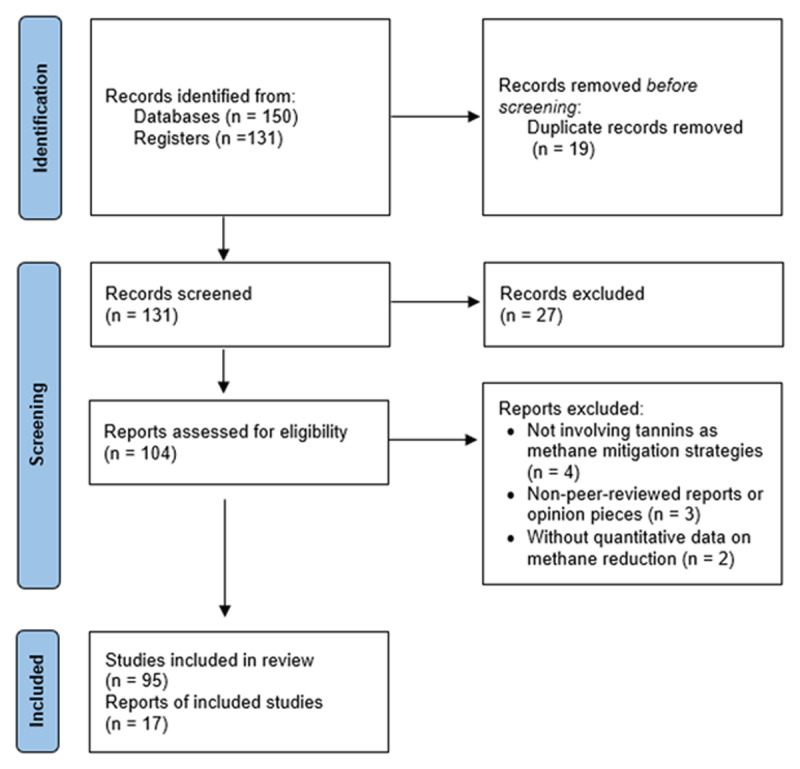
PRISMA flow diagram summarizing the identification, screening, eligibility assessment, and inclusion of articles.

Out of the first search, 150 articles were found. Once duplicates were removed, 131 records remained. Title and abstract screening excluded 27 articles that did not meet the inclusion criteria. After that, the eligibility of 104 articles was evaluated, and 95 that met the inclusion criteria were included in this review. Among them, 17 articles were used as primary data sources, while the others served as supplementary references in the discussion. The overall selection process is presented in the PRISMA flow diagram (Figure 1).

### 2.5. Data extraction and synthesis

Data relevant to the selected study were systematically extracted and categorized according to specific research themes. The main variables registered were: 1) source and type of tannins (hydrolyzable vs. condensed); 2) mechanisms of action on rumen microbiota and pathways to mitigate methane; 3) Effects on the fermentation processes in the rumen: The characteristics of microbial populations, volatile fatty acids (VFA), and nitrogen utilization; 4) Synergistic effects attributed to tannins in combination or other dietary interventions like biochar, essential oils, and probiotics. 5) Negative effects on nutrition, variety of tannin sources, and microbial adaptation. Narrative data synthesis was applied due to differences in study design and reporting. Where possible, studies were classified by tannin type, plant source, dosage, and animal species to enable a semi-quantitative comparison. The authors reached a consensus through discussions to resolve conflicting findings.

## 3. Conceptual Framework

**Figure 2.**
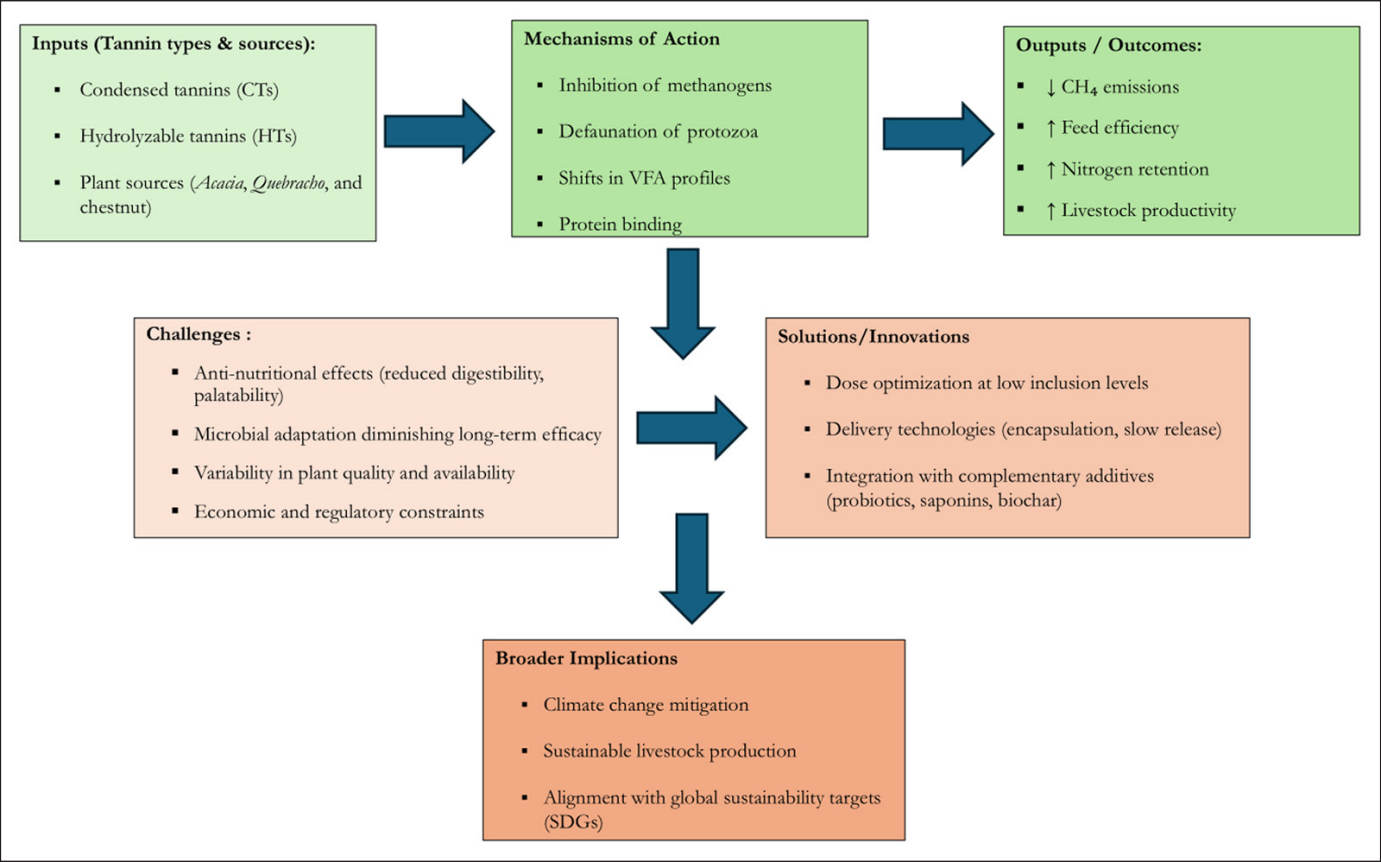
Conceptual framework of the role of tannins in enteric methane mitigation.

A conceptual foundation for the tannin-based methane-reduction technique was laid. Tannins exert diverse effects on rumen fermentation, methane production, and animal productivity. To integrate these various aspects, we suggest a conceptual framework (Figure 2) that links the chemistry of tannins, their interactions with the rumen microbiome, the consequent fermentation shifts, and the nexus between livestock productivity and sustainability. On the biochemical level, the interactions of condensed tannins (CTs) and hydrolyzable tannins (HTs) occur with proteins, carbohydrates, and microbial enzymes present in the diet. These interactions not only diminish the availability of substrate for methanogenesis but also alter the VFA (volatile fatty acid) profiles. From a microbiological point of view, tannins are substances that primarily inhibit methanogenic archaea while simultaneously suppressing protozoa that harbor endosymbiotic methanogens; therefore, the hydrogen used to form methane is reduced.

Tannins, while acting as nutritional aids, serve as nitrogen protectors by blocking dietary proteins from degrading in the rumen, thereby augmenting the intestinal flow of undegraded protein. Moreover, the situation could flip if tannin intake is uncontrolled, compromising not only fiber digestibility but also animal performance; hence, proper management of dosage is of utmost importance.

From a systems viewpoint, tannin supplementation results in reduced enteric methane emissions, increased feed efficiency, and decreased nitrogen excretion, all of which are consistent with the sustainability objectives of reducing greenhouse gas emissions and improving resource efficiency. If such supplementation is accompanied by complementary mitigation strategies (such as probiotics, lipids, and biochar), climate-smart livestock production of tannins offers the ultimate synergistic potential.

This framework allows for a fresh perspective on the tannin’s role in methane mitigation as interrelated and not as separate biochemical events through the interconnected processes that link the rumen and reveal their various aspects.

## 4. Types and Characteristics of Tannins

Tannins are complex plant secondary metabolites that have attracted interest primarily for their potential to reduce methane emissions from ruminants. Their makeup and activity are determined by factors such as plant species, country of origin, and environment, including season, temperature range, and soil conditions, as well as processing, which may alter the structural properties of the compounds [12, 13, 15]. Tannins can be further classified into two categories: condensed tannins (CTs) or hydrolysable tannins (HTs). Flavonols and HT-derived glucose esters, including ellagic or gallic acids or HT, were observed to be effective against the rumen microbiota and to reduce methane production [10, 16].

**Figure 3.**
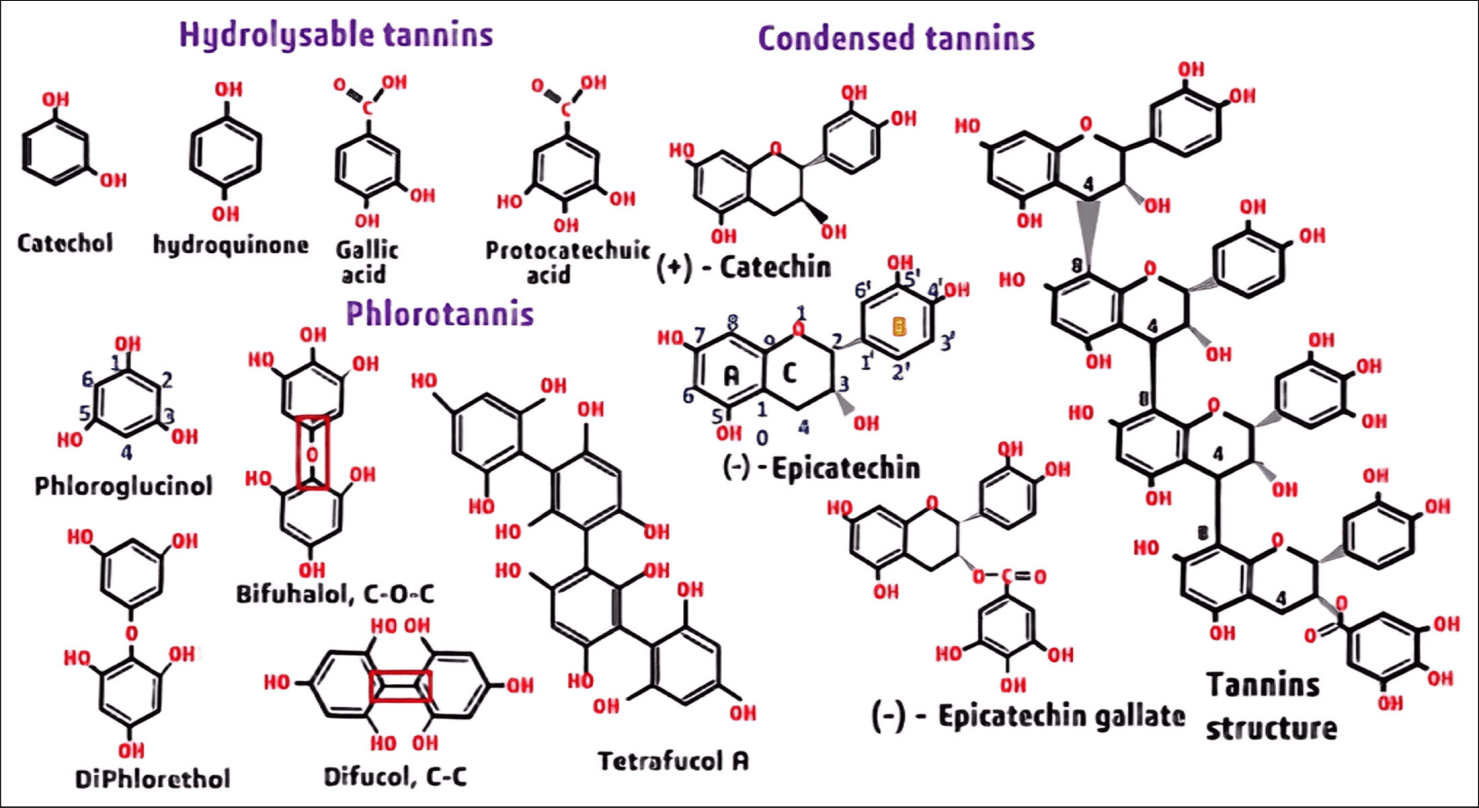
Chemical structure of hydrolysable and condensed tannins (adapted from Aboagye and Beauchemin (adapted from [18]).

The chemical structure of tannins characterizes polyphenols with several hydroxyl (-OH) groups attached to the aromatic rings (Figure 3). Condensed tannins, or proanthocyanidins, interact with rumen bacteria to limit methane emissions. They bind dietary proteins, lowering degradation rates and thereby benefiting livestock fed high-rumen-degradable-protein diets [11]. The efficacy of condensed tannins is determined by their molecular structure and weight, with lower-molecular-weight tannins displaying stronger inhibition [17, 18]. Plant sources rich in CTs include *Schinopsis balansae, Acacia* spp., *Trifolium repens* L., and leguminous forages such as *Lotus corniculatus* and *Onobrychis viciifolia* [19, 20, 21].

Hydrolysable tannins are either gallotannins or ellagitannins, which hydrolyze into ellagic acid and gallic acid [22]. Generally, HTs have lower molecular weights, higher solubility, and degrade more easily than CTs. Hydrolysable tannins provide anti-inflammatory and anti-cancer benefits and contribute to methane reduction by altering microbial fermentation [23, 24, 25, 26]. In addition, in the rumen, HTs can decrease methane production by altering microbial fermentation, although the impact is less pronounced than that of CTs [3, 22]. Important plant sources of HTs include pomegranate (*Punica granatum*) [23], chestnut wood (*Castanea sativa*) rich in vescalagin and castalagin [24], and carob pods (*Ceratonia siliqua*) [25].

Therefore, what distinguishes CTs and HTs is degradability, structural stability, and the intensity of biological effects in the rumen. In ruminants, tannins are important because they bind food proteins, reducing their breakdown and the amount of substrate available for methanogenesis. Tannins offer a viable approach to reducing methane emissions in ruminants through multiple mechanisms of action. However, careful application is necessary to maximize benefits while minimizing adverse effects on livestock health and productivity. Optimal dosing, structure-activity, and the interactions of tannins as agro-industrial by-products with other dietary components should be analyzed to increase their use in sustainable livestock production, either alone or as plant combinations and extracts.

## 5. Mechanisms of Action

### 5.1. Enteric methane production in ruminants

An anaerobic environment is suitable for feed fermentation through the activities of diverse populations of microorganisms in the rumen. The fermentation process is a key feature of ruminants’ specialized digestive system, allowing them to digest plant materials that would otherwise be indigestible. The rumen, the largest chamber of the ruminant stomach, is rich in various microorganisms, including bacteria, protozoa, and fungi, that work together to break down complex carbohydrates, proteins, and other nutrients into smaller, more readily absorbable products of digestion [4]. A wide variety of nutrients are degraded during this process, resulting in the production of volatile fatty acids (VFAs), carbon dioxide (CO_2_), and hydrogen (H_2_). Moreover, the amount of hydrogen can significantly impact the fermentation process, either preventing it or allowing it to proceed. On the one hand, methanogens are microorganisms that transform H_2_ and CO_2_ into CH_4_ via methanogenesis; on the other hand, they also stabilize the fermentation process [27, 28]. However, this conversion can remove 2–12% of energy from gross energy intake and significantly contributes to greenhouse gas emissions [18, 29, 30].

Methane production in the rumen is a multi-layered biochemical process driven by diverse microbial populations. This process is closely associated with synergistic interactions among diverse microbial groups. Cellulolytic bacteria (e.g., *Fibrobacter succinogenes* and *Ruminococcus flavefaciens*) are important in degrading dietary fiber into smaller molecules for fermentation [31]. Besides cellulolytic bacteria, hydrogen is also produced by the fermentation of organic matter consumed by rumen protozoa in the rumen, and some protozoan species are notably engaged in a symbiotic relationship with methanogens, where methanogens consume the hydrogen produced by protozoa to support methanogenesis [32, 33]. Anaerobic fungi will accelerate further fiber degradation, thereby indirectly providing additional hydrogen. *Methanobrevibacter* spp. are the microorganisms that are usually in the majority in ruminants and are extremely specialized in producing methane from H_2_ and CO_2_ through methanogenesis, while *Methanosarcina* spp. can use methanol and methylamines as substrates for methane production. This suggests that methanogens in the rumen employ different metabolic strategies [27, 34].

Various factors, such as feed composition, animal type, and management practices, determine the quantity of methane produced in the rumen. Fiber-rich feeds, like forage, lead to increased methane emissions because fiber fermentation produces more hydrogen, which is the main substrate for methanogenesis, while diets rich in concentrates may indirectly contribute to methane production through the activity of protozoa. Grain-based feeding promotes propionate formation, which competes with hydrogen for methanogenesis and lowers emissions [33, 35, 36]. Moreover, the type of forage is also an important factor; red clover silage is less associated with methane emissions than grass silage [37]. The overall methane emissions from cows are higher than those from sheep and goats. This is mainly because they have a greater rumen retention time, leading to increased microbial fermentation [3, 38, 39].

To put it simply, enteric methane production is a complex biochemical process that involves interactions among rumen microorganisms, the host’s metabolic functions, and feed materials. On the one hand, methanogenesis may help stabilize the balance of rumen fermentation processes, and on the other hand, it may decrease feed energy utilization and produce greenhouse gases. Gaining a deep insight into these processes is a prerequisite for developing ruminant methane-reduction strategies.

### 5.2. Mechanisms of action of tannins in methane mitigation

The anti-methanogenic effect of tannins works through several interrelated mechanisms, including inhibition of methanogenic archaea, reduction of protozoa populations, changes in volatile fatty acid (VFA) profiles, and modification of protein degradation and nitrogen utilization in the rumen. Each pathway reduces the hydrogen available for methanogenesis, thereby reducing methane emissions (Figure 2).

#### 5.2.1. Inhibition of methanogens

Condensed tannins (CTs) and hydrolyzable tannins (HTs) directly inhibit methanogenic archaea by binding to their cell membranes and enzymes, thereby suppressing hydrogenotrophic methanogenesis [10, 22]. Methane can be significantly reduced *in vitro*, even at low tannin concentrations [40, 41], whereas *in vivo* studies yield less consistent responses due to dietary variations and microbial adaptation [3, 42]. Therefore, although methanogen inhibition is the main mechanism, its long-term effectiveness under practical feeding conditions remains unclear.

Tannins can directly reduce methane formation by decreasing the number and activity of methanogens, as well as by limiting hydrogen availability (indirectly) [10, 43]. Its effectiveness depends on the source, type, and dose of tannins, such as HT, which can reduce methanogens by 11.6%, and a combination of HT and CT, which can reduce methanogens by 28.6%. Moreover, environmental factors are highly influential, with phenolics affecting the adhesion and dynamics of hydrogenotrophic methanogens, thereby altering the ecology of rumen microbes [44]. Although tannins from leguminous plants have high antimethanogenic potential, their long-term effects may be reduced as microbes adapt.

#### 5.2.2. Reduction of protozoa populations

Protozoa play a significant role in methane formation because they harbor symbiotic methanogens and produce hydrogen (H_2_) during fermentation, which serves as the main substrate for methanogenesis [45, 46]. The addition of tannins reduces the protozoa population and can indirectly reduce methane emissions [22, 47, 46]. This effect depends on the dosage: moderate levels suppress protozoa without disrupting fiber digestion, whereas excessive levels disrupt rumen function [47, 48].

Bhatta et al. [49] reported in an *in vitro* study that HT supplementation reduced protozoa numbers by 12.3%, while combined HT and CT treatment reduced them by 36.2%. Other research has indicated that tannin-rich diets, including those containing quebracho tannin, can significantly reduce protozoa numbers and methane production [48]. In summary, in addition to restricting hydrogen transfer to methanogens, a decrease in protozoal populations might also disrupt their symbiotic relationships, thereby providing a significant indirect route for methane reduction.

#### 5.2.3. Shifts in volatile fatty acid (VFA) profiles

Tannins alter rumen fermentation by changing the VFA proportions, decreasing the acetate fraction, and increasing the propionate fraction [48, 50]. Propionate production is increased because it functions as a hydrogen absorber. The most relevant change in methanogenesis that reduces methane production is the withdrawal of hydrogen gas (H_2_). *In vitro* tests have shown that alterations in VFAs are almost equivalent [33, 51, 52]; nonetheless, prolonged *in vivo* experiments have indicated that microbial adaptability might reduce the effects of these changes to some extent [10].

Alterations in microbial communities are influenced by several factors, such as dosage, tannin type, and diet, producing different effects; even among tannins, some can create acetate-to-propionate ratios that are more advantageous than others. With modifications in the VFA profile, methane is not the only compound reduced; total energy utilization in ruminants may also increase, making the use of tannins via the microbial population a non-therapeutic option for anti-methanogenic effects.

#### 5.2.4. Protein binding and nitrogen utilization

Tannins form complexes with dietary proteins, reducing protein degradation and decreasing the availability of substrates that support methanogenesis. The effect of this protection is a consistent increase in nitrogen retention, an increase in the flow of undigested protein to the intestine, and a reduction in urinary nitrogen excretion, as reported in tropical plants containing tannins [11, 18, 48]. In addition to reducing methane formation, improved nitrogen use efficiency also improves nutrient efficiency in ruminants.

However, the benefits of protein savings depend on the type of tannin, concentration, and method of administration. Tannins will increase nitrogen use efficiency at the right level, but protein digestibility, feed intake will decrease, and ultimately animal performance will be impaired if the concentration is excessive [53]. Achieving the right balance is essential to maximize the anti-methanogenic benefits while minimizing potential anti-nutritional effects.

### 5.3. Overall mechanisms of action

Methane emissions from ruminant livestock are modified by tannins through different but interconnected routes. Tannins directly inhibit methanogenic archaea, reduce protozoal counts, and change the VFA (volatile fatty acid) profiles. In addition, on the one hand, tannin-protein complexes provide nitrogen very efficiently, while on the other hand, they protect proteins from ruminal degradation, thereby reducing methane formation by restricting substrates that support its production.

The degree and the duration of these effects are determined by the type, concentration, and source of tannins, as well as the feeding conditions. Moderate levels of tannins can increase rumen efficiency and reduce methane emissions, whereas high levels have the opposite effect. That is, there is a decline in nutrient digestibility and animal performance in such situations. Therefore, prudent management of tannin supplementation is essential to prevent its antimicrobial potential from becoming detrimental to animal productivity.

## 6. Efficacy Across Systems

The application of tannins in the diet as a strategy to reduce enteric methane emissions has been extensively tested across various systems, including *in vitro* and *in vivo* animal trials (Table 1). Cross-study effectiveness is supported by the considerable potential revealed in some experiments; however, it is also variable, depending on the study design, tannin type, and livestock species used.

**Table 1.** Overview of experimental studies using tannin-based feed additives to reduce methane emissions in ruminants.

Tannin source	System/Species	Dosage	Methane reduction	Reference
Acacia tree bark extract	*In vitro*	2% DM	15–25%	[48]
Leaf powder	*In vitro*	1.5–2.5% DM	16%	[69]
Tanniferous extracts	*In vitro*	1.2% DM	18%	[59]
Legume extracts	*In vitro*	12–30 gm/kg DM	15%	[60]
*G. biloba* extracts	*In vitro*	1.6% DM	53%	[54]
*O. viciifolia* extracts	*In vitro*	40, 80 gm/kg of DM	19.4, 16.1%	[61]
*C. papaya* extracts	*In vitro*	15 mg/0.25 gm DM	34%	[55]
Chestnut leaves extracts	*In vitro*	24 mg/gm DM	28%	[56]
*Leucaena leucocephala* extracts	Crossbred Heifers	240 gm/kg DM	11.14%	[62]
Extracts of Quebracho + chestnut	Nellore bulls	0.7 gm/kg DM	17.3%	[92]
Chestnut and Quebracho extracts	Beef Cattle	15 gm/kg DM	6%	[11]
*Delonix regia* seed meal	Beef Cattle	27 gm/kg DM	16%	[63]
*Artocarpus heterophyllus, Azadirachta indica* and *Ficus benghalensis*	Sheep	72 gm/kg DM	24%	[64]

### 6.1. In vitro studies

The inhibition of methanogenesis by feed additives rich in tannins has been confirmed *in vitro* studies. Nevertheless, *in vitro* research reports that the effects depend on dosage, plant source, and the extraction method used. Generally, methane reduction ranged from 15% to 50%, though it could be even higher. The Ginkgo biloba extract produced the greatest methane reduction, more than 50% at 1.6% of feed DM [54], while extracts from Carica papaya leaf and chestnut reduced methane production by 34% [55] and 28% [56], respectively. More moderate reductions were observed in *Acacia* bark at around 15–25% [57], leaf powder at 16% [58], tannin extract at 18% [59], legume extract at 15% [60], and *Onobrychis viciifolia* at around 16–19% [61].

*In vitro* studies show a consistent pattern: even at relatively low levels of addition, tannins exhibit a measurable inhibitory effect on methanogenesis, often exceeding the magnitude observed in *in vivo* studies. However, the chemical composition of different plant species plays a role in determining effectiveness. Thus, overall, *in vitro* studies confirm that tannins can suppress methane production under controlled and significant conditions. However, some studies report that the magnitude of reduction in *in vitro* tests tends to overestimate effectiveness in the field, at 30–50%, because these tests do not capture the adaptive capacity of rumen microbes or the physiological effects of the host. Therefore, *in vitro* results should be interpreted with caution and verified *in vivo*, although they are valuable for screening candidate plants.

### 6.2. In vivo studies

Although the effects produced are generally smaller than *in vitro* tests, *in vivo* tests (direct tests on livestock) have proven that tannins can reduce methane emissions. The reduction depends on the source of tannins, dosage, and ruminant species, ranging from 6 to 24%. For example, supplementation with *Leucaena leucocephala* extract in crossbred cows reduced methane emissions by 11% [62], while feeding Delonix regia seed meal to beef cattle achieved a 16% reduction [63]. The efficiency of tannin-rich plant mixtures is plainly demonstrated in the mixture of *Artocarpus heterophyllus, Azadirachta indica*, and *Ficus benghalensis*, which reduced methane in sheep by 24% [64].

It is a matter of fact that, in some situations, higher doses are being applied, and thus, the case of chestnut and quebracho extracts in beef cattle is not exactly the same, where a dose of 15 gm/kg DM reduced methane by only 6% [11]; nonetheless, lower doses were more effective. This reversal of outcomes emphasizes the quality of tannin effectiveness, which depends on the conditions and is determined by diet and microbial adaptation.

Tannins, however, have consistently inhibited methanogenesis, but the extent of this inhibition depends on the system employed. *In vitro* tests tend to exaggerate the tannins’ potential, as long-term microbial adaptation and host physiological responses are absent. Although *in vivo* results are more moderate, they provide a realistic picture of their applicability in production systems. These differences require caution when directly generalizing laboratory findings to agricultural settings. However, there is evidence that tannins may be among the factors contributing to reduced methane production, and that their effectiveness varies in specific contexts.

Various discrepancies across studies indicate that the effectiveness of tannins depends on the combination of several factors, including the type of tannin, its amount, the type of feed, the animal species, and the rumen microbial community. Rumen microbes, however, may slowly overcome the suppressive effects of tannins; still, this is a major problem in the long run.

### 6.3. Comparisons of condensed and hydrolyzable tannins

The comparison, hence, illustrates the dissimilarities between the mechanisms of the two kinds of tannins: hydrolyzable and condensed. Hydrolyzable tannins (HTs) and condensed tannins (CTs) have remarkably different chemical structures, stabilities, and interactions with the rumen ecosystem, which ultimately determine their adaptability to the enteric methane-emissions-reduction process. HTs are more susceptible to hydrolysis by acids, bases, and rumen enzymes because HTs are esters of gallic acid or ellagic acid bound to a glucose nucleus. Thus, simple phenolic compounds are released and have a relatively short effect in the rumen. On the contrary, CTs are very resistant to microbial breakdown, since they are composed of flavonoid chains such as catechin and epicatechin, and can therefore provide a steadier, longer-lasting effect.

There are also substantial differences in the interaction between these two types of tannins with feed substrates. HTs can bind to proteins, but their binding is weak and reversible, providing little protection against rumen protein degradation and only a small impact on hydrogen availability. Conversely, CTs form strong, stable complexes with proteins and structural carbohydrates, thereby reducing rumen protein degradation and ammonium formation. This decreases accounts not only for reductions in hydrogen reserves but also for an increase in NUE (Nitrogen Use Efficiency). In addition, the effects of these two types of tannins on microbial populations also differ. HTs are known to disrupt fiber digestion when present in the rumen at high levels, since this type of tannin mainly prevents fibrolytic bacteria from growing rather than methanogens or protozoa. Rumen HTs might be more selective and, as a result, more favorable for essential methanogenic functions in the rumen than CTs. The reason is that CTs disrupt symbiosis, hindering hydrogen transfer between the species and, consequently, leading to a decline in methane production. The distinction lies in the fermentation pattern.

Changes in VFA composition are usually due to phenolic metabolites of HTs, which are toxic and are accompanied by a decline in total VFA concentration. On the other hand, CTs regularly direct fermentation towards propionate production, which then serves as a hydrogen sink for microorganisms. Such hydrogen flow diversion increases competition with methanogenesis and, therefore, decreases methane production without losing energy efficiency.

Overall, HTs produce their anti-methanogenic effect primarily through direct toxicity to methanogens, but their impact tends to be moderate, temporary, and potentially detrimental to digestibility when added at high levels. In contrast, CTs work through diverse and complementary mechanisms, providing more effective, consistent, and sustainable reductions in enteric methane emissions (when administered at low to moderate levels).

Other findings indicate that combined supplementation with CTs and HTs provides a greater synergistic effect than separate use. This may be due to complementary or exclusive mechanistic actions on fermentation and methanogenesis. For example, supplementation with HT and CT has been shown to be more effective at suppressing methanogenesis than HT alone, without excessively interfering with fermentation [65].

### 6.4. Microbial adaptation and long-term effects

As antimethanogenic agents, tannins exhibit limited long-term effectiveness due to microbial adaptation. The rumen microbial community undergoes structural shifts, enriching tannin-resistant populations and activating detoxification pathways, thereby reducing the inhibitory effect of prolonged feeding [42]. Diet composition also affects this response: the consumption of easily fermentable carbohydrates mostly deactivates tannins, whereas grass-based diets increase tannins’ overall potency.

Microbial tolerance and enzymatic degradation are among the adaptation mechanisms. Some bacteria, like *Klebsiella pneumoniae*, can hydrolyze tannins or even use them as a nutrient source. For example, the former can achieve 98% degradation of tannic acid within 72 h via tannase activity and, thus, serves as the sole source of carbon and energy [66]. Tannase, which is widely distributed in bacteria, fungi, and yeast, breaks down tannins into glucose and gallic acid, thereby reducing their antimicrobial properties [48]. Other rumen microflora members digest tannin-protein complexes, as in situ studies demonstrate the rapid disappearance of free and bound tannins in 24 h, namely *Streptococcus gallolyticus, Lonepinella koalarum*, and *Selenomonas ruminantium* [67]. This adaptation decreases tannins’ capacity to improve feed efficiency, inhibit methanogens, and consequently reduce methane emissions.

Further evidence from long-term trials supports this pattern. Battelli et al. [10] highlight a decrease in tannin-sensitive microbial populations, which interferes with methane suppression, while Baihaqi et al. [3] state that the initial reduction in methane gradually decreases during prolonged feeding. Tannins selectively inhibit certain microbial groups, allowing resistant populations to survive and recover [20].

Research is being conducted on complementary techniques to retain long-term efficiency. Encapsulation might hinder microorganisms’ access to tannins; using other bioactive substances alongside it could enhance the effect, and alternating supplementation could minimize adaptation pressure [20]. Notably, Yogianto et al. [43] showed that bamboo leaf tannins can selectively inhibit methanogenic archaea while preserving beneficial microbes, which paves the way for an effective method of targeted methane reduction.

## 7. Implementation Challenges

### 7.1. Practical challenges of using tannins as feed additives

Even though the application of tannins as feed additives has been found reasonable at one time, there always remain constraints on consistency and long-term utility in relation with some factors. High levels of tannins can reduce feed intake, decrease nutrient absorption, and lower protein digestibility. Nevertheless, continuous supplementation may lead to bacterial adaptation, resulting in a gradual loss of anti-methanogenic activity. In addition, the effects of tannins on ruminant performance and methane emissions remain uncertain, as they depend on the types, sources, and concentrations of tannins.

To address these limitations, encapsulation technology, which enables controlled, slow-release of tannins in the rumen, where both methane suppression and nutrient utilization occur, has been proposed as a potential solution. According to Adejoro et al. [20], lipid-protected tannins were more effective in reducing methane emissions in sheep than unprotected extracts.

In a similar manner, Ibrahim and Hassen [68] reported that in Merino sheep, encapsulated tannins reduced methane emissions by 21.7%, whereas non-encapsulated forms reduced them by 19.1%, and neither treatment caused any drop in digestibility.

Several studies have shown that tannin efficacy can be enhanced when combined with other feed additives. The highest reduction of methane was observed when tannins and saponins were used together [69], whereas Besharati et al. [70] reported a synergistic effect with the combination of tannins and either saponins or lipids. Khoa et al. [71] reported that methane production was significantly reduced during co-digestion with biochar, although biochar did not impair digestibility. Authors noted the porous structure of biochar, which traps aggregates and mitigates the growth of methane-producing bacteria. Likewise, Yogianto et al. [72] reported that combining tannin-rich shrubs with tannin supplements increased protein supply, supported growth, and decreased methane production. Both tannins and fats, as well as essential oils, have been shown to inhibit methane production and enhance the availability of proteins and enzyme activities [73, 74].

The formulation of the diet and the right dosage are also imperative. Alkahtani et al. [75] suggest that the source of tannins should be balanced with other nutrients to support digestion, as proper supplementation increases protozoa and rumen fermentation [46]. It has been reported that optimal tannin levels can reduce methane production without affecting livestock growth [67], and to maximize nutrient use, selecting tannin composition is pivotal [48]. Furthermore, the specific reactions of different organisms should also be considered. Qi et al. [22] emphasized gradual adaptation and controlled dosing; by contrast, Mueller-Harvey et al. [76] suggested potent but non-toxic concentrations, and Ibrahim and Hassen [77] argued for continuous monitoring of animal health and methane emissions to develop better strategies.

Within a framework, the impact of tannins on methane reduction is significantly greater. Getiso et al. [73] note that combining tannins with other methods of reduction can enhance their performance, whereas Hristov et al. [78] point to dietary changes, such as adding more concentrates, to alter fermentation. The choice of tannin-bearing plants has also been shown to lower methane emissions by 7–9%, including species such as *Acacia mearnsii* and *Onobrychis viciifolia* [79].

In practice, tannins are only effective when their source, dosage, and method of administration are aligned with the animal’s nutritional regimen. The synergistic effects of encapsulation technology, innovative diet formulations, and additives, along with the proper monitoring and adjustments, not only allow tannins to act as the productivity boosters and the backbone of sustainable livestock systems.

### 7.2. Economic considerations

The impact of tannins on beef systems is always a function of their biological efficacy and economic viability. This underscores the need to consider the socio-economic aspects of tannin supplementation, particularly in resource-poor agricultural systems.

The incorporation of tannin-containing feed additives has numerous economic impacts, mainly due to their ability to inhibit methanogens, thereby regulating rumen microbial populations and altering fermentation dynamics by making hydrogen unavailable for methane formation [3, 80]. Besides mitigating climate change, reducing methane emissions can enhance livestock performance through increased energy retention [10, 11, 75]. Tannins are known to modulate rumen fermentation and improve nutrient absorption, thereby increasing animal productivity and health [48, 70]; hence, they are often considered a feeding strategy favorable to the environment. Tannin also aligns with sustainable agriculture by reducing environmental impacts while improving animal welfare and the quality of production. [46]. Consequently, not only can livestock producers obtain a more eco-friendly and sustainable farming system by concentrating on sustainable sources of tannins and incorporating tannin-rich crops into feeding strategies, but they can also do so with the help of the latter.

Nonetheless, when weighing the pros and cons of tannin supplementation, one must consider the numerous hurdles that arise. Most of the time, the acquisition and processing of tannin extracts drive up costs even more than conventional feed additives [81]. Moreover, high levels of addition can have adverse effects, leading to productivity losses that may equal or exceed the economic gain from reducing methane [82]. As a result, the use of tannins is highly context-dependent and influenced by several factors, including the local availability of tannin-rich feed, the type of production system, the market price of commercial extracts, and the applicable policy framework or incentives.

The use of tannin supplements by farmers is greatly influenced by economic considerations, particularly cost, availability, and scalability. Economic factors such as cost, availability, and scalability greatly influence farmers’ use of tannin supplements. The factors in Table 2, along with methane reduction potential, offer a comparison of selected tannin sources. The implementation of tannin-rich feed sources in livestock systems is driven by their accessibility and affordability. In this regard, grape pomace has been found to reduce methane emissions by 10–11%, with farmers incurring no or very little cost [83]. The tropical legume *Calliandra calothyrsus*, which contains high tannins, can be cultivated by farmers and has been reported to enhance the performance of small ruminants while reducing methane production [84]. Likewise, the leaves of *Acacia mearnsii*, a plant popular in agroforestry systems, have been shown to reduce protozoa and methanogens without affecting digestibility, making it a cost-effective local option [18]. Indigofera, another local legume that competes with the highest-protein low-cost legume, meanwhile, is affected by the presence of antinutritional factors necessitating proper processing or dosage adjustment when given to animals [85].

**Table 2.** Comparison of selected tannin sources with respect to cost, scalability, and methane mitigation potential.

Tannin Source	Cost / Availability	Scalability	Methane Mitigation Effectiveness	Reference
Grape pomace	Low cost; by-product of wine industry; readily available in viticulture regions	High, depends on agro-industrial by-product streams	Reduces CH_4_ by ~10–11% in small ruminants without added costs	[83]
*Calliandra calothyrsus*	Low cost; can be cultivated by smallholder farmers	Moderate to high; suited to tropical smallholder systems	Improves animal health and performance; reduces methane emissions	[84]
*Acacia mearnsii* leaves	Readily available in agroforestry systems; minimal costs	High in areas where agroforestry is practiced	Suppresses protozoa and methanogens; no adverse effect on digestibility	[18]
*Indigofera* sp.	Locally available legume with high protein content; low cost	Moderate; requires management of antinutritional factors	Potentially reduces CH_4_ but effectiveness depends on dose and processing	[85]

Local sources of tannins, like grape pomace, *Calliandra*, and *Acacia*, which are abundant, provide small herds with a way to reduce methane emissions that are both eco-friendly and economical from a practical point of view. Nevertheless, scientists admit that further research and development are needed in the areas of costs and benefits, additional strategies, and actual agricultural conditions to carry out adoption analyses.

### 7.3. Regulatory considerations

Tannins have different legal statuses in almost every region. In Europe, restrictions on certain tannin extracts, such as quebracho, allow their use in animal feed, but their market is limited because they cannot be classified as methane-reducing additives. In the US, although conventional tannin sources are generally considered safe, they do not meet the requirements for recognition as methane-reducing agents. The regulatory environment in Asia varies greatly from country to country. India and China are more tolerant of tannins than the rest of the world, allowing the use of feed additives, while other countries maintain strict prohibitions. The absence of harmonized regulations hinders the commercialization of tannins specifically for methane reduction. Future policy developments, especially in the context of carbon-neutral livestock production, may create clearer pathways for approval and adoption.

## 8. Research Gaps and Future Directions

Understanding of tannin-based interventions in livestock continues to evolve (particularly in ruminants), but critical knowledge remains limited. There is no certainty yet as to whether rumen taxa or genes enable bacteria to tolerate or degrade tannins, how quickly adaptation occurs, or whether such adaptation can reduce desired outcomes (such as protein protection or methane reduction). Most existing studies are short-term or descriptive [86, 87]. Future research should include longitudinal *in vitro* and *in vivo* adaptation studies, combined with metabolomics, shotgun metagenomics, meta-transcriptomics, and targeted enzyme assays (e.g., tannase) to evaluate functional effects. Isolation and characterization of tannin-tolerant strains, along with the study of phage dynamics, are also recommended.

Second, responses vary by breed and species (goats, sheep, cattle) and are influenced by host genetics, rumen morphology, chewing habits, and prior exposure to tannin-rich forages [70, 88]. Controlled crossbreed trials assessing productivity, methane emissions, rumen microbiota, intake, digestibility, and nitrogen excretion, integrated with host genotyping and behavioral/salivary studies, are needed to clarify host-microbiome interactions and breed-specific differences.

Third, tannin bioactivity depends on chemical type, degree of polymerization, and co-compounds. Many studies fail to characterize tannins precisely, complicating cross-study synthesis. Future studies should quantify condensed versus hydrolysable tannins, molecular weight, subunit composition, and distinguish between whole-forage sources and purified extracts. Dose-response matrices are essential to define beneficial versus anti-nutritional thresholds.

Fourth, the mechanistic understanding of trade-offs between methane mitigation and fiber intake/digestion remains limited [10, 89]. Future work should use isotope tracer studies, H_2_ balances, methanogen qPCR, *in vitro* gas generation, and *in vivo* CH_4_ detection to distinguish direct anti-methanogenic effects from indirect consequences on rumen fermentation. Strategies to maintain fiber-degrading taxa or employ enzyme co-supplementation should be explored.

Fifth, long-term effects on immunity, reproduction, longevity, and product quality are underexplored [90, 91]. Eventually, the longitudinal herd studies will follow up on the trial results by examining gut barrier function and oxidative stress biomarkers, and by assessing interactions with other phytochemicals (saponins, terpenes) to assess overall health benefits.

Finally, this technology still suffers from the aforementioned factors, such as variations in tannin granule size, digestibility, and the different combinations of feed additives used. A thorough evaluation of the economic aspects, delivery methods (encapsulation, pellet extraction, binding agents), agronomic evaluation of tannin-rich feeds, and life-cycle analysis must be conducted as part of the various assessments required to facilitate practical implementation.

## 9. Conclusions

Natural feed additives, particularly tannins, have been proven effective in ruminants for reducing enteric methane emissions and improving nutrient intake. The latter occurs through methanogen inhibition, protozoa suppression, fermentation shifts, and enhanced nitrogen efficiency. Tannins are less troublesome when administered at a proper dosage, at a controlled consumption rate, and with proper microbial adaptation. Optimized inclusion strategies, encapsulation technologies, and synergistic use with other bioactive compounds are among the promising solutions that could help sustain efficacy without compromising animal performance. Therefore, the use of tannins is seen as a practical and sustainable means of reducing greenhouse gas production and improving climate-smart livestock systems.

## Data Availability

The data presented in this study are available from the corresponding author upon reasonable request.

## List of abbreviations

BRIN, National Research and Innovation Agency; CH_4_, methane; CO_2_, carbon dioxide; CTs, condensed tannins; DM, dry matter; GHG, greenhouse gas; H_2_, hydrogen; h, hours; HTs, hydrolysable tannins; kg, kilogram; mg/kg, milligram per kilogram; gm/kg DM, gram per kilogram of dry matter; N_2_O, nitrous oxide; NUE, nitrogen use efficiency; PRISMA, Preferred Reporting Items for Systematic Reviews and Meta-Analyses; qPCR, quantitative polymerase chain reaction; spp., species; VFA, volatile fatty acid.
